# Transforming Breast Imaging: A Narrative Review of Systematic Evidence on Artificial Intelligence in Mammographic Practice

**DOI:** 10.3390/diagnostics15172197

**Published:** 2025-08-29

**Authors:** Andrea Lastrucci, Nicola Iosca, Yannick Wandael, Angelo Barra, Renzo Ricci, Jacopo Nori Cucchiari, Nevio Forini, Graziano Lepri, Daniele Giansanti

**Affiliations:** 1 Department of Allied Health Professions, Azienda Ospedaliero-Universitaria Careggi, 50134 Florence, Italyioscan@aou-careggi.toscana.it (N.I.); wandaely@aou-careggi.toscana.it (Y.W.); barraa@aou-careggi.toscana.it (A.B.); riccire@aou-careggi.toscana.it (R.R.); 2 Breast Imaging Unit, Department of Radiology, Azienda Ospedaliero-Universitaria Careggi, 50134 Florence, Italy; norij@aou-careggi.toscana.it; 3 Dipartimento di Medicina e Chirurgia, Università degli Studi di Perugia, Piazzale Settimio Gambuli, 1, 06129 Perugia, Italy; nevio.forini@unipg.it; 4 Unità Sanitaria Locale Umbria 1, Via Guerriero Guerra 21, 06127 Perugia, Italy; graziano.lepri@uslumbria1.it; 5 Centre TISP, Istituto Superiore di Sanità, 00161 Rome, Italy

**Keywords:** mammography, artificial intelligence, breast cancer

## Abstract

**Background**: Breast cancer is still the most common type of cancer worldwide. Advances and the global use of artificial intelligence (AI) have opened up new opportunities to improve diagnostic accuracy and optimize breast cancer screening. This review summarizes the findings from systematic reviews to assess the current situation of AI integration in mammography. **Methods**: A total of 28 systematic reviews were included and analyzed using a standardized narrative checklist to assess the impact of AI on mammography imaging. Bibliometric analysis and thematic synthesis were used to assess trends, evaluate the performance of AI in different modalities and identify challenges and opportunities for clinical implementation. **Results and Discussion**: AI technologies show an overall performance comparable to radiologists in terms of sensitivity and specificity, especially when integrated with human interpretation to detect breast cancer in mammography. However, most studies are retrospective, which raises concerns about their generalizability to real-world clinical settings. Key limitations include potential dataset bias—often stemming from the over-representation of specific imaging equipment or clinical environments—limited ethnic and demographic diversity, the lack of model explainability that hinders clinical trust, and an unclear or evolving legal and regulatory framework that complicates integration into standard practice. **Conclusions**: AI has the potential to transform mammography screening, but its integration into the real world requires prospective validation, ethical safeguards and robust regulatory oversight. Coordinated international efforts are essential to ensure that AI is used safely, fairly and effectively in breast cancer diagnostics.

## 1. Introduction

### 1.1. Breast Cancer Epidemiology and Clinical Features

Breast cancer is the most frequently diagnosed cancer worldwide. In 2020, 2.3 million new cases were reported globally, accounting for 1 in every 8 cancers diagnosed and making it the most common cancer overall, according to data from the International Agency for Research on Cancer (IARC) [1]. That same year, breast cancer caused approximately 685,000 deaths, making it the leading cause of cancer death among women and the fifth leading cause of cancer mortality overall [1].

Although the disease predominantly affects women, male breast cancer occurs in about 1% of cases [2]. Incidence rates are highest in high-income countries—such as Australia, Western Europe, and North America—but are increasing in many low- and middle-income countries (LMICs), where demographic transitions and lifestyle changes are shifting cancer patterns [1,3].

From a clinical perspective, breast cancer is biologically and histologically heterogeneous, arising most frequently in ductal or lobular epithelium, and is categorized into molecular subtypes based on hormone receptor and HER2 status [4]. These subtypes are key in determining prognosis and guiding personalized treatment approaches.

Despite significant advances in early detection and therapeutic strategies—especially in high-resource settings—breast cancer remains a major global health challenge, with substantial disparities in access to care and survival outcomes [1,3,5].

### 1.2. Evolution of Mammographic Imaging

The origins of mammographic imaging can be traced back to the early 20th century. In 1913, German surgeon Albert Salomon was the first to systematically study breast tissue using X-rays, comparing radiographic findings with histological samples from over 3000 mastectomies. His pioneering work laid the foundation for the use of X-ray imaging in breast cancer diagnosis [6].

However, it was not until the 1950s and 1960s that mammography began to emerge as a distinct clinical tool. The development of dedicated mammography units, lower-dose radiation techniques, and improved film-screen combinations marked a major shift from general radiography to breast-specific imaging. The introduction of xeromammography in the 1960s, although later abandoned, represented an early effort to enhance image clarity and lesion detection [7].

By the 1970s and 1980s, mammography had become more widely adopted in clinical settings, particularly after studies began to demonstrate its efficacy in early cancer detection. Landmark trials such as the Health Insurance Plan (HIP) study in New York showed that routine mammographic screening could significantly reduce breast cancer mortality among women aged 50 and older [8].

The transition to digital mammography in the late 1990s and early 2000s represented a major technological leap. Unlike analog systems, Full-Field Digital Mammography (FFDM) offered enhanced contrast resolution, image storage flexibility, and the potential for advanced image processing and telemedicine applications [9]. These benefits were validated in large comparative trials, including the Digital Mammographic Imaging Screening Trial (DMIST), which demonstrated the superior sensitivity of digital mammography in women under 50 and those with dense breasts [9].

More recently, the advent of digital breast tomosynthesis (DBT)—also known as 3D mammography—has further refined diagnostic accuracy. By acquiring multiple low-dose images at different angles, DBT reconstructs a pseudo-3D representation of the breast, improving lesion visibility and reducing recall rates [10].

Today, mammographic imaging is a cornerstone of breast cancer screening and diagnosis. Its evolution reflects a continuous pursuit of greater sensitivity and specificity, driven by technological innovation and a deeper understanding of breast cancer biology.

### 1.3. Artificial Intelligence Integration in Mammography

This long-standing trajectory has laid the groundwork for the integration of artificial intelligence (AI) into mammographic workflows—a development aimed at optimizing interpretation, reducing diagnostic variability, and addressing the growing demand for radiological services.

The integration of artificial intelligence (AI) into mammographic imaging represents a promising frontier aimed at enhancing breast cancer detection and diagnosis. Initial explorations of AI in mammography began several decades ago, inspired by the potential of computer-aided detection (CAD) systems developed in the late 20th century [11].

Early CAD systems were designed to assist radiologists by highlighting suspicious areas in mammograms, thus acting as a “second reader.” Despite improving sensitivity in some cases, these first-generation tools often suffered from high false-positive rates, limiting their widespread adoption [12].

With advances in machine learning and, more recently, deep learning techniques, AI applications in mammography have experienced significant renewed interest. These advanced models are capable of analyzing complex imaging patterns beyond human visual perception, offering the potential to improve diagnostic accuracy, reduce inter- and intra-observer variability, and streamline radiologists’ workflow. Consequently, AI has shown promise not only as a second reader but also as a tool for prioritizing cases and assisting in risk stratification [13].

However, the gradual integration of AI tools into clinical mammography has raised important questions and discussions within the medical community. These concern the need for rigorous clinical validation, the transparency of AI decision-making processes, and their seamless integration into existing diagnostic pathways to ensure safety, reliability, and acceptance by practitioners [14].

### 1.4. Current Challenges and Opportunities in AI-Driven Breast Cancer Screening

The COVID-19 pandemic has significantly disrupted routine breast cancer screening programs worldwide, leading to delayed diagnoses and increased challenges for healthcare systems [15]. In response to these challenges, there has been a rapid acceleration in the adoption and development of artificial intelligence (AI) applications in mammography. AI-driven tools have been explored to support remote screening workflows, prioritize high-risk cases, and manage increased workloads, thereby helping to mitigate the impact of service disruptions [16,17]. This surge in interest highlights AI’s potential role not only in improving diagnostic accuracy but also in enhancing the resilience and efficiency of breast cancer screening programs during healthcare crises.

Recent advances in artificial intelligence (AI) have introduced powerful techniques such as machine learning (ML), artificial neural networks (ANNs), and deep learning (DL), which are revolutionizing many fields including medicine [18]. These AI methods enable automated decision-making by learning from complex and large datasets, simulating aspects of human cognition through interconnected processing layers. In the medical domain, these approaches have demonstrated promising potential to enhance diagnostic speed, accuracy, and efficiency across various applications [18], with potential also in breast cancer screening. Specifically, AI tools can support mammographic image analysis by improving lesion detection and classification, facilitating earlier diagnosis and personalized patient care. Nevertheless, the integration of AI into clinical workflows still faces important challenges. Key among these is the need for the explainability and transparency of AI systems, which are essential to build clinician trust, address ethical concerns, and comply with regulatory standards [19]. Overcoming these barriers is crucial for the widespread adoption of AI-assisted mammography and for fully realizing AI’s potential to improve breast cancer detection outcomes.

Simultaneously, the evolution of breast cancer imaging encompasses a wide range of conventional and emerging modalities, with AI positioned as a transformative component in this landscape [20].

These developments highlight both exciting opportunities and complex obstacles, underscoring the critical need for the comprehensive synthesis and evaluation of existing evidence to guide safe and effective AI implementation in mammography.

### 1.5. Framing the Research: Key Questions and Aims of the Narrative Review

The rapid advancement of artificial intelligence (AI) technologies has opened new horizons in mammographic breast cancer screening, yet a comprehensive understanding of their impact remains essential. Several broad and fundamental questions arise in this evolving field:

First, what is the current state of AI applications in mammography across clinical, technical, and research domains?

Second, how does the integration of AI influence diagnostic accuracy, workflow efficiency, and healthcare delivery in breast imaging?

Third, what are the main barriers and facilitators affecting the adoption and implementation of AI technologies in breast cancer screening programs? And finally, what ethical, legal, and social implications emerge from the use of AI in this context?

Addressing these questions is critical to guide future research, optimize clinical applications, and support informed policy decisions for the safe and responsible integration of AI into breast cancer detection and management.

Aim

The purpose of this study is to conduct a narrative review to comprehensively explore the role of artificial intelligence (AI) in mammographic breast cancer screening. This review aims to aggregate and synthesize findings from existing systematic reviews in order to provide a consolidated and high-level understanding of how AI is currently applied within mammography. By identifying and analyzing key themes and trends emerging from these reviews, this study seeks to highlight significant advancements such as improvements in diagnostic accuracy, workflow efficiency, and clinical outcomes and examine the challenges related to implementation, standardization, and ethical considerations.

Specific aims:

- Trend analysis: Map recent trends in the scientific literature on AI in mammography, highlighting growth areas, publication volume, and thematic shifts using bibliometric insights.
- Themes and categorization: Identify and organize the main themes emerging from existing systematic reviews, focusing on AI applications that enhance breast cancer detection and diagnostic processes in mammography.
- Opportunities and challenges: Analyze the opportunities and challenges reported in the literature, including benefits related to diagnostic performance and clinical workflow, alongside barriers encountered in real-world adoption and integration.

## 2. Materials and Methods

This work was conducted as a narrative review, aiming to collect and synthesize existing systematic reviews and meta-analyses on the use of artificial intelligence (AI) in mammography, with a specific focus on practical applications in breast cancer screening. This review followed the ANDJ narrative checklist.

A targeted literature search was performed on PubMed, Scopus and the Web of Science using a composite query designed to identify relevant reviews. The search string is reported in Box 1.

Box 1 **Used search keys.**

(“artificial intelligence” OR “machine learning” OR “deep learning” OR “convolutional neural networks,” “transformers,” or “radiomics”)AND (“mammography” OR “breast cancer screening”)

The search retrieved articles without date restrictions, although most of the studies included were published within the last ten years.

During the selection process, we prioritized reviews that investigated the actual implementation and clinical application of AI methods in mammography—such as diagnostic support, lesion detection, triage strategies, and risk stratification. Reviews with only general, theoretical, or purely mathematical overviews of AI, without reference to mammographic practice, were excluded.

Each study was evaluated through a structured quality checklist based on prior methodological guidance. For each included review, six key parameters were assessed:

- N1: Clarity of the study’s rationale.
- N2: The appropriateness of the design.
- N3: The transparency of the described methods.
- N4: Clarity in presenting results.
- N5: Coherence between results and conclusions.
- N6: The disclosure of conflicts of interest.

Parameters N1 to N5 were graded on a 5-point scale (1 = very poor, 5 = excellent), while N6 was assessed binary: “Yes” if conflicts of interest were clearly disclosed or “No” if not.

Only studies meeting the following criteria were included in the final synthesis:

- A “Yes” rating for N6 (conflict of interest disclosure).
- A score of 3 or higher on each of the five graded parameters (N1–N5).

The selected reviews were then analyzed through a focused investigation with particular care, for example, on the following:

- The types of AI algorithms applied (e.g., CNNs, ensemble learning),
- The clinical tasks and workflow integration,
- The datasets and validation strategies used,
- The reported strengths, weaknesses, and evidence gaps.

This process enabled a comprehensive overview of the current state of AI applications in mammography, highlighting both converging evidence and areas requiring further investigation.

Each study was independently evaluated by two reviewers (AL and DG). In cases of disagreement, a third reviewer (either GL or NF) acted as adjudicator to reach consensus.

The anonymized assessment, seen in Table S1, listing the included studies is reported in the Supplementary Materials.

Potential sources of bias were considered at multiple stages of the review process. Selection bias was minimized by applying predefined eligibility criteria and performing independent dual screening. Assessment bias was reduced through the structured checklist and the adjudication process in cases of reviewer disagreement.

A total of 28 studies were included in the study as they matched the focus of this review [21,22,23,24,25,26,27,28,29,30,31,32,33,34,35,36,37,38,39,40,41,42,43,44,45,46,47,48].

## 3. Results

### 3.1. Trend Analysis

To better understand the growth trajectory and maturity of artificial intelligence (AI) research applied to mammography, we conducted a comparative trend analysis across several adjacent clinical fields. The aim of this analysis is twofold: first, to characterize the specific evolution of AI-focused mammography literature over time, and second, to contextualize this growth by comparing it with broader trends in AI research in radiology, oncology, and breast cancer at large.

This comparison is important to establish whether mammography AI research is following a unique path or whether it mirrors more general patterns seen in medical AI literature. By identifying shared or divergent trends, we gain insight into how mammography sits within the wider AI–healthcare landscape—whether as a lagging, leading, or aligned subfield.

To do so, we employed four targeted literature searches (Search Keys 1–4, shown in Box S1 in the Supplementary Materials), which represent increasing levels of generalization:

- Search Key 1 focuses on mammography and AI (our core interest).
- Keys 2, 3, and 4 expand the scope to radiology, oncology, and breast cancer, respectively, still retaining AI as a central theme.

Using Search Key 1 from Box S1, on the core focus of this review on artificial intelligence (AI) applied to mammography, we identified a total of 1748 studies published between 1989 and 2024 on PubMed. Among these, 221 were reviews or systematic reviews, indicating a mature body of synthesized evidence in this niche area.

Publication trends show a clear acceleration over the past decade, with 1312 studies (about 75%) published in the last 10 years and a substantial 1042 studies (60%) in just the last 5 years. Notably, this means that nearly 80% of the last decade’s output was concentrated in the most recent half—a strong indicator of recent intensified research activity.

Several factors likely contribute to this surge. Technologically, advances in deep learning architectures, the increasing availability of large annotated mammographic datasets, and improvements in computational resources have made AI applications in mammography more feasible and effective. Systemically, many health systems worldwide have prioritized digital transformation initiatives, fostering the development and deployment of AI tools for diagnostic imaging.

Importantly, the COVID-19 pandemic appears to have acted as a catalyst accelerating this growth. During the pandemic, radiology departments faced critical workforce shortages, backlog pressures, and triage complexities, all of which heightened interest in AI-driven solutions to maintain diagnostic throughput and resilience. Moreover, the pandemic’s push towards digital health and remote care created a conducive environment for AI innovation and evaluation, particularly in imaging fields.

To contextualize these findings, we compared this focused trend with broader AI research in related clinical domains using other key search strings from Box S1:

- For Key 2 (*“artificial intelligence” AND “radiology”*), a vast total of 32,854 publications since 1960 were found, with an overwhelming 87.9% (28,864) published in the last 10 years and 74.0% (24,317) in the last 5 years. The most recent 5 years alone represent about 84.3% of the publications produced in the preceding 5-year period (from 10 to 5 years ago), demonstrating a pronounced clustering of research activity.
- For Key 3 (*“artificial intelligence” AND “oncology”*), there have been 43,252 publications since 1961, with 81.7% (35,337) from the last decade and 65.7% (28,423) from the last 5 years. The most recent 5 years account for approximately 80.5% of the output of the prior 5-year interval, reflecting a similarly strong acceleration.
- For Key 4 (*“artificial intelligence” AND “breast cancer”*), a more focused yet significant dataset of 7292 publications since 1986 was identified, with 83.3% (6077) from the last 10 years and 66.8% (4872) from the last 5 years. The most recent 5 years make up about 80.2% of the preceding 5-year output, highlighting a consistent pattern of concentrated growth in AI research specific to breast cancer.

Overall, these comparisons reveal a strikingly consistent pattern: across the domains of radiology, oncology, breast cancer, and mammography-focused AI research, roughly 80% to 84% of the research output in the last five years corresponds to the research produced in the preceding five-year period. In other words, about four-fifths of the publications from the last decade were generated in just the most recent half of that time span.

This convergence around the 80% mark highlights a strong clustering of research activity in the most recent years, reflecting a common, rapid acceleration of AI-driven studies across these related but distinct fields.

Mammography-focused research (Key 1), despite being smaller in total volume, aligns clearly with this pattern, illustrating its role as an emerging but integral component within the broader AI and cancer imaging research ecosystem.

This shared trajectory underscores how advances in computational power, machine learning methods, and the availability of large-scale annotated datasets, combined with systemic incentives such as digital health policies and clinical needs, are collectively fueling this surge.

Additionally, the COVID-19 pandemic likely acted as a catalyst across all domains, increasing the urgency of and resource allocation to AI research aimed at improving diagnostic capacity, workflow resilience, and remote care options during a period of unprecedented healthcare strain.

In summary, these aligned trends demonstrate that AI research in mammography is not isolated but part of a broader, interlinked expansion of AI applications in medical imaging and oncology—all experiencing a similarly steep growth curve driven by technological innovation and healthcare challenges.

### 3.2. The Integration of AI in Mammography: The Focus on Emerging Themes and Technological Innovations

A total of 28 articles were included in this study as they matched the focus of this narrative review [21,22,23,24,25,26,27,28,29,30,31,32,33,34,35,36,37,38,39,40,41,42,43,44,45,46,47,48]. An analysis of the studies was conducted with a particular focus on the integration of AI technology in mammography, which is summarized in Table 1. A brief supplementary summary of the systematic reviews included can be found below.

The study by Abu Abeelh and Abuabeileh [21] reports that AI in screening mammography has the potential to improve diagnostic accuracy, reduce false-positive results and reduce radiologists’ workload. Although generally comparable or even superior to traditional methods, challenges such as false-positive results and variability in subgroup performance require further research.

The study by Yoon et al. [22] found that standalone AI in screening digital mammography performs comparably or better than radiologists, particularly in reader studies and digital breast tomosynthesis. AI showed higher sensitivity but lower specificity, highlighting both its potential and current limitations.

The review by Zeng et al. [23] is focused on the frequency and characteristics of AI errors in reading screening mammography and it reported that AI errors primarily involve false positives and false negatives, with their frequency varying by algorithm threshold and version. Other error types like localization and technical errors are under-reported, limiting the full understanding of AI limitations.

The review by Freeman et al. [24] found that most AI systems were less accurate than radiologists in large, retrospective studies, and none outperformed double reading. While smaller studies showed promise, there is insufficient evidence to support AI replacing radiologists in breast cancer screening without further prospective evaluation.

The study by Laws et al. [25] highlights significant gaps in mammography datasets used for AI development, including limited global representation, poor documentation, and a lack of demographic transparency. Only a small fraction of datasets are publicly accessible, and most lack diversity data critical for equitable AI advancement.

The study by Branco et al. [26] found that AI algorithms in mammography screening generally perform comparably to radiologists and improve diagnostic accuracy when used alongside them. AI can support radiologists, especially less experienced ones, by enhancing lesion detection and overall evaluation accuracy.

In the review by Schopf et al. [27], it is reported that AI models using only mammography images predict future breast cancer risk more accurately than traditional clinical risk tools, with median area under the receiver operating characteristic curve (AUC) of 0.72 versus 0.61. Adding clinical data to AI models provided minimal to no additional predictive benefit.

The systematic review by Avanzo et al. [28] examined the application of AI in medical imaging, with a section dedicated to mammography. The review highlights that AI technologies support breast cancer detection and characterization by enhancing diagnostic accuracy, reducing false negatives, and facilitating precision medicine. Advanced methods such as deep convolutional neural networks (CNNs), clustering algorithms, and classifiers significantly improve lesion detection across various imaging modalities, including digital mammography, contrast-enhanced spectral mammography (CESM), and tomosynthesis.

The systematic review by Rentiya et al. [29] highlights the evolving role of AI in breast cancer detection and treatment, emphasizing its potential to improve diagnostic accuracy and efficiency beyond traditional methods like computer-aided detection. While AI integration shows promise, the review underscores the importance of addressing challenges related to data quality, validation, ethics, and regulation to fully realize its benefits in clinical practice.

The review by Batchu et al. [30] summarizes evidence on AI’s utility in mammography, highlighting its potential to reduce false positives, increase sensitivity (up to 91%), and assist in both diagnosis and risk prediction. Despite heterogeneity across studies and the need for more prospective research, AI shows promise in reducing radiologist workload and improving diagnostic performance.

The study by Xavier et al. [31] demonstrates that deep learning (DL)-based triage can significantly reduce radiologist workload—by over 68%—while maintaining high sensitivity (93.1%) in detecting breast cancer, supporting its potential as an effective tool in screening workflows. However, high heterogeneity across studies and systems highlights the need for standardized implementation frameworks and more prospective validation.

The systematic review and meta-analysis by Hickman et al. [32] found that stand-alone machine learning algorithms in screening mammography can match or exceed human reader performance (AUC 0.89 vs. 0.85), with the potential for significant workflow optimization through triage. However, the results are largely retrospective and limited in scope, emphasizing the urgent need for prospective validation in real-world conditions to confirm these promising results.

The study by Liu et al. [33] confirms the high diagnostic accuracy of ML methods in mammography, especially convolutional neural networks (CNNs), which outperformed other models with a pooled AUC of 0.974, a sensitivity of 96.1% and a specificity of 95.0%. These results emphasize the great potential of CNNs in breast cancer screening workflows, although prospective, real-world studies are needed to validate their clinical impact.

The review by Anderson et al. [34] emphasizes that while AI algorithms for mammography screening show a modest improvement in diagnostic accuracy, especially when combined with radiologists, the current evidence is limited by retrospective designs and methodological weaknesses. Few studies have used robust real-world results, emphasizing the urgent need for high-quality, prospective external validation to establish the true clinical benefits of AI.

The study by Bhalla et al. [35] analyzes DL studies for breast cancer detection at mammography, focusing on reproducibility and best practice. Most studies showed a high risk of bias due to unrepresentative patient selection. Only four studies met appropriate quality standards, using models such as ResNet and RetinaNet, with the best AUC values of 0.93–0.95. Combining AI with radiologist assessments improved accuracy and specificity. However, none of the studies provided meaningful explanations beyond lesion localization or assessed AI–radiologist interaction in real clinical settings.

The review by Goh et al. [36] highlights a critical gap in the current DL literature on mammography: despite promising diagnostic performance (e.g., AUCs up to 0.955 with combined AI-radiologist readings), most studies lack reproducibility and explainability. An overwhelming 96% were flagged for a high risk of bias, largely due to non-representative datasets. Few studies train robust, transparent models, and none assess real-world AI–radiologist interaction or offered interpretable insights beyond localization.

The study by Gurmessa and Jimma [37] examines explainable AI (XAI) methods for breast cancer diagnosis using mammography and ultrasound images. Most studies highlighted issues with datasets as a key research gap. The relationship between accuracy and explainability remains unclear, and real-world assessments of XAI reliability are lacking. Overall, the review emphasizes the need for further research to improve explainability, dataset quality, and confidence in XAI systems for clinical use.

The review by Hussain et al. [38] examines how DL and AI are used to predict breast cancer risk by analyzing imaging, genetic, and clinical data. Traditional models rely mostly on demographics and history, but AI offers more personalized predictions. AI techniques like natural language processing can improve risk assessment and support better screening and management. The review provides a clear overview of current progress and future possibilities in AI-based breast cancer risk prediction.

The review by Oh et al. [39] analyzed radiomics—a technique used for extracting detailed data from medical images—to differentiate benign from malignant breast lesions. Results from MRI, mammography, and ultrasound showed high accuracy, with sensitivities around 79–92% and specificities around 81–85%. Radiomics shows promise as a non-invasive diagnostic aid for breast cancer, but biopsy remains the definitive standard due to current technological and study limitations.

The study by Hussain et al. [40] examines the applications of DL, radiomics, and radio genomics in digital breast tomosynthesis (DBT), an advanced 3D mammography technique rapidly replacing traditional 2D mammography. The study highlights how these AI-driven approaches enhance breast cancer detection and patient stratification by linking imaging features with genetic information. The review provides insights into the current landscape and future directions of DL, radiomics, and radio genomics in DBT imaging for early breast cancer diagnosis.

The review by Madani et al. [41] examines how DL improves breast cancer detection using different imaging methods, including mammography. Early detection is vital but current manual screening is slow, expensive, and error-prone. AI, especially DL, offers automated, more accurate analysis to support radiologists. The paper discusses the pros and cons of various imaging techniques, summarizes recent AI research, and highlights key datasets for training models.

The review by Gardezi [42] examines the use of ML and DL for breast cancer diagnosis using mammography data. Traditional methods are limited, while DL shows significant improvements despite challenges such as data scarcity and high computational costs, which are partially addressed by data augmentation and higher computational power. The detection of masses in dense breast tissue is more challenging compared to calcifications.

The study by Yassin et al. [43] reviews the current state of CAD systems for breast cancer using different imaging modalities. The review focuses on ML-based classifiers and image types used in CAD systems and highlights advances and areas for future research to improve the objectivity and efficiency of breast cancer detection and diagnosis.

The review by Singh and Singh [44] examines the use of breast thermography for the early detection of breast cancer. Recent advancements in artificial neural networks and ML have improved diagnostic accuracy by improving image analysis steps such as segmentation and classification. Despite some challenges, such as false positives, the integration of numerical simulations and newer ML methods promises more reliable real-time breast cancer screening in the future.

The study by Sorin et al. [45] focuses on DL applications in contrast-enhanced mammography (CEM), a newer imaging modality that improves breast cancer diagnosis. The DL models focused on lesion classification, detection and segmentation and achieved a wide range of accuracy (AUC 0.53 to 0.99). Attention-based models showed high accuracy (~88–89%), and combining DL with radiomics or radiological input further improved performance. Most studies, while promising, are retrospective and emphasize the need for prospective clinical validation to confirm DL’s real-world effectiveness in CEM.

The relevance of AI-enhanced mammography is further reinforced by a recent systematic review and meta-analysis by Ciurescu et al. [46], who evaluate the diagnostic and prognostic value of AI-assisted mammography in conjunction with the Systemic Immune–Inflammation Index (SII). Their work explores the synergy between imaging biomarkers and systemic markers, indicating new horizons for integrated AI-driven assessment in breast cancer.

While not focused exclusively on mammography, the systematic review by Rostami et al. [47] discusses AI’s role in identifying cancers of unknown primary, reinforcing the cross-cutting impact of AI in oncologic imaging and diagnostics. Their findings highlight how radiomics and AI-guided workflows contribute to narrowing diagnostic uncertainty in highly complex cases.

Finally, Bennett et al. [48] present an updated systematic review on breast cancer screening, emphasizing not only the evolving evidence base but also the implications for national screening guidelines. Although AI is not the central focus, the review acknowledges the increasing relevance of technology-assisted strategies in improving early detection, reinforcing the system-level integration of AI into preventive health efforts.

Table 1 presents the common themes that emerged from the included studies concerning the application of AI in mammographic practice.

Table 2 goes deep into technological implementations and details on AI applications. Numerous studies have explored and confirmed the potential of artificial intelligence (AI) to improve the early diagnosis of breast cancer through mammography. AI is primarily applied to assist the reading of radiographic images, reduce the workload of radiologists, improve diagnostic sensitivity and specificity, and reduce false positives that cause unnecessary recalls and anxiety in patients [21,22,30].

*Diagnostic Performance and Types of Algorithms*

AI developed for mammography typically employs machine learning (ML) and deep learning (DL) models, with the widespread use of convolutional neural networks (CNNs) for automated image analysis [33,42]. CNNs demonstrate superior performance compared to other techniques such as artificial neural networks (ANNs) or support vector machines (SVMs), reaching sensitivity over 95% and AUC (area under the ROC curve) values close to 0.97 [33].

Most studies evaluate AI algorithms either as standalone systems or in combination with clinical assessment by radiologists [22,26,34]. Meta-analytic results indicate that standalone algorithms can match or surpass radiologists’ sensitivity, though often with slightly lower specificity, while the human–machine combination tends to improve overall accuracy [22,34].

*Reduction in False Positives and Workload*

A key feature of AI is its ability to significantly reduce false positives, with reductions reported up to 69% [30]. This has a strong clinical impact as it lowers unnecessary recalls, improving the cost-effectiveness of screening. Additionally, deep learning-based systems have been studied as triage tools capable of excluding low-risk cases, reducing the radiologist’s workload by up to 68% without compromising sensitivity, which remains above 90% [31].

*Datasets and Generalizability*

A critical issue highlighted is the quality and representativeness of datasets used to train and validate AI models. Many datasets are private, with poor or absent demographic descriptions, limiting the generalizability and fairness of AI across diverse populations [25]. Moreover, variability in algorithm performance based on population and clinical setting underscores the need for external and multicenter validations, which remain infrequent [26,34].

*Errors and Technical Challenges*

AI is not free from errors, with frequent false positives and false negatives depending on decision thresholds and algorithm versions [23]. Some reviews also report a lack of transparency and difficulty in explainability of AI decisions, which is an important limitation for clinical adoption and operator trust [35,37].

*Clinical Integration and Governance*

The literature emphasizes the complexity of integrating AI into mammographic screening workflows due to factors such as technological interoperability, staff training, regulation, and ethical and legal issues [27,36]. Governance frameworks based on evidence standards, continuous monitoring, and strategies to build trust are necessary for safe and sustainable AI adoption [36].

*Multidisciplinary Applications and Future Perspectives*

Beyond mammography, AI is also applied to complementary imaging modalities such as digital breast tomosynthesis, magnetic resonance imaging, and ultrasound, often integrating radiomics and radio genomics techniques for more accurate diagnosis and personalized risk stratification [38,39,40]. The combined use of clinical, genomic, and imaging data through deep learning models promises to transform breast cancer management, from early diagnosis to prognosis and treatment.

### 3.3. Opportunities and Challenges for Applying Artificial Intelligence in Mammography

The incorporation of AI in mammography is transforming breast cancer screening and diagnosis. Whereas AI has huge potential for enhanced diagnostic accuracy, streamlined processes, and personalized care, its clinical deployment is qualified by issues related to data representativeness, regulations, and credibility.

One of its most promising advantages is improved diagnostic performance. Some studies demonstrate that deep learning models, namely convolutional neural networks (CNNs), are comparable to, if not better than, experienced radiologists. For example, Liu et al. reported that CNN-based models achieve an AUC value of 0.974, outperforming conventional ML algorithms such as support vector machines and artificial neural networks (ANNs) [33]. Similarly, Yoon et al. found that independent AI models performed better than radiologists on DBT alone, achieving a higher AUC (0.90 vs. 0.79), although specificity was slightly lower [22]. These results reflect the promise of AI to improve the early detection of cancer, a key factor in patient outcomes.

AI may also reduce radiologists’ workload, a major problem in high-volume screening programs. Several systematic reviews reported that the use of AI-assisted triage systems can reliably rule out 68% of low-risk mammograms, with diagnostic sensitivity exceeding 90% [31,32]. Improving workflow in this way not only reduces the burden on medical staff but also increases efficiency and potentially reduces report turnaround time.

Personalized risk prediction is another new possibility. Standard models typically use clinical and demographic information—such as breast density, family history and age—to predict a woman’s risk of breast cancer later in life. However, AI models based directly on mammography images offer better predictive ability [27,28].

In addition, AI can support a radiologist’s assessment if it is used as a complement, not a substitute. Castelo Branco et al. and Anderson et al. concluded in their systematic reviews that performance regularly improved when human interpretation and AI were used together and that improvements in AUC ranged from 0.02 to 0.13 [26,34].

In addition, AI is regularly applied to other advanced imaging modalities. Sorin et al. show that CNN-based algorithms have already achieved AUCs of 0.99 or higher when retrospectively analyzing CEM [45]. The future of AI-assisted mammography beyond routine 2D mammography seems clear, especially for more complex imaging conditions such as DBT and CE mammography.

Despite these encouraging developments, significant challenges remain. The biggest obstacle is the lack of validation and generalization beyond training and test data. As highlighted by Freeman et al. and Anderson et al., most studies to date are retrospective and based on highly curated data that are not rich in natural diversity and complexity [24,34]. In the absence of large prospective studies, it is difficult to assess performance in routine clinical practice, where data heterogeneity and variability in surgery cannot be avoided.

The representativeness of the dataset is another related and equally important problem. Laws et al. explain that only 12% of the datasets used for training AI are related to ethnicity or ethnicity and the data are mainly from high-income areas such as Europe, North America and East Asia [25]. Such non-diversification may lead to biases in the AI training algorithms, which could then perform worse in unrepresented populations.

Ethical, legal and regulatory issues also limit the use of AI. The review by Goh et al. has identified several main problems, including inadequate frameworks, uncertainties regarding liability for errors in algorithms and the protection of patient data privacy [36]. In addition, there is the difficulty of establishing universal standards for the safety and performance of AI models. In most countries, regulatory standards for the use of medical image AI are either underdeveloped or unevenly enforced. Finally, the heterogeneity of technologies contributes to the overall complexity of the field. As documented by Batchu et al. and Madani et al. the use of different AI architectures, imaging modalities, and validation methods hampers direct comparison across studies and prevents meaningful synthesis of results [30,41].

Overall, AI holds great potential for mammographic imaging, from improved diagnostic accuracy to personalized screening protocols and reduced clinician burden. However, realizing these benefits comes with significant hurdles in terms of data diversity, explainability, approvability and clinical validation. The potential integration of AI into breast screening soon will depend on interdisciplinary collaboration between clinicians, data scientists, regulators and ethicists. Only through multilateral collaboration can AI be transitioned from an experimental promise to routine clinical practice in a safe, fair and trustworthy manner.

**Figure 1:**
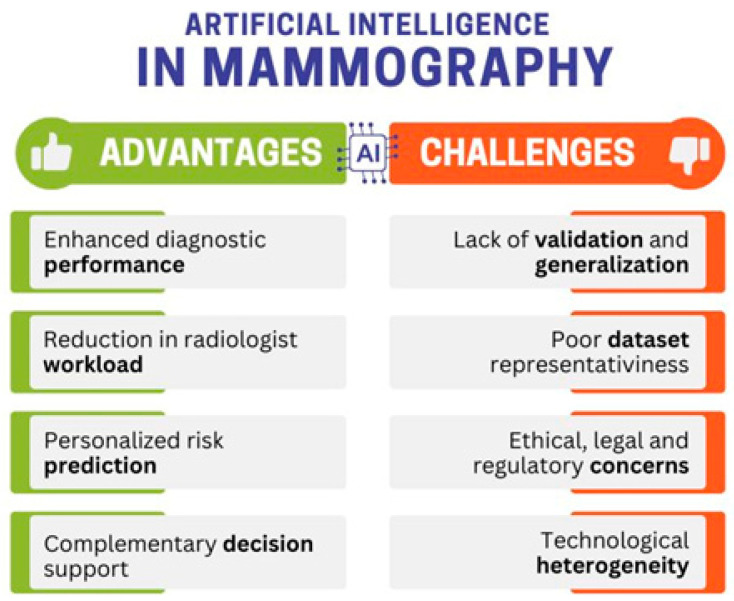
Graphical summary of key opportunities and challenges in applying AI to mammography imaging.

Figure 1 presents a graphical summary of the key challenges and opportunities previously described regarding the application of AI technology in mammography imaging.

## 4. Discussion

### 4.1. Current Trends and Evidence Synthesis on AI Applications in Mammography

In recent years, the application of artificial intelligence (AI) in mammography has attracted growing attention from researchers, clinicians, and healthcare stakeholders alike. This surge reflects the promise of AI to enhance breast cancer detection, improve diagnostic accuracy, and alleviate the workload on radiologists. The field has experienced a rapid expansion in scientific publications, fueled by technological advancements, the availability of large, annotated imaging datasets, and increased computational capabilities.

A search for studies combining the terms “mammography” and “artificial intelligence” reveals an impressive growth in publications over the last three decades, with over 75% of the research produced in just the past ten years and 60% in the last five years alone. This trend mirrors the broader momentum in AI applications within radiology and oncology, where similar exponential growth patterns are observed.

Several factors have contributed to this accelerated development. Advances in deep learning algorithms and imaging technology, alongside global digital health initiatives, have paved the way for innovative AI tools in breast cancer screening. The COVID-19 pandemic further acted as a catalyst, compelling healthcare systems worldwide to adopt digital solutions for maintaining diagnostic services amid workforce challenges and social distancing requirements.

To provide a comprehensive synthesis of the rapidly growing but fragmented literature, a narrative review was conducted. This review aimed to integrate existing systematic reviews and meta-analyses on AI applications in mammography, thereby overcoming the heterogeneity and methodological variability of individual studies. By analyzing 28 systematic reviews, the narrative review offers a higher-level perspective that consolidates diverse data sources and highlights both consensus and gaps in current knowledge.

The added value of this narrative review lies in several key aspects: first, its capacity to assess the robustness of evidence across varied populations, AI models, and clinical settings, thus offering guidance for future research priorities and clinical implementation strategies; second, its identification of methodological strengths and limitations that inform best practices for AI validation; third, its comprehensive evaluation of AI’s impact on radiologist workload and diagnostic performance; and finally, its critical examination of challenges related to algorithm explainability, data bias, and regulatory frameworks

This multi-dimensional analysis is crucial for translating AI innovations into safe, effective, and equitable clinical tools.

Key findings from the synthesized systematic reviews and meta-analyses confirm that AI holds significant potential to improve diagnostic sensitivity, reduce radiologists’ workload, and optimize clinical workflows. For instance, Abu Abeelh and Abuabeileh [21] and Yoon et al. [22] report that AI systems can match or even outperform radiologists in breast cancer screening, though limitations remain concerning false positives and false negatives. Zeng et al. [23] further emphasize that AI errors are mostly related to false-positive and false-negative rates, which are influenced by threshold settings and different algorithm versions.

Nevertheless, multiple reviews underscore the pressing need for further prospective and standardized studies to validate AI’s clinical effectiveness in real-world settings. Freeman et al. [24] and Anderson et al. [34] caution against considering AI as a substitute for radiologists without robust prospective validation, highlighting that most existing evidence derives from retrospective studies with notable methodological limitations. Similarly, Hickman et al. [32] advocate for prospective clinical trials to confirm the promising results generated by machine learning algorithms.

Another important finding pertains to workload reduction for radiologists. Studies such as Batchu et al. [30] and Xavier et al. [31] demonstrate that AI systems—especially those based on deep learning—can effectively triage low-risk mammograms, significantly decreasing the volume of images requiring manual review without compromising diagnostic sensitivity.

A recurrent theme in the literature is the synergistic combination of AI and human radiologists. Many studies indicate that integrating AI outputs with clinical evaluation improves diagnostic performance compared to either AI or radiologists alone [35,36]. However, Goh et al. [36] and Gurmessa and Jimma [37] point out challenges related to the explainability and reproducibility of AI models, which are crucial for clinical acceptance and patient safety. The lack of transparency and biases due to non-representative datasets remain significant hurdles [35,36].

Data quality and representativeness are also critical concerns. Laws et al. [25] highlight significant gaps in available datasets, especially regarding global representativeness and demographic transparency, which are essential for developing equitable and generalizable AI models.

Advances have also been observed in AI applications to advanced mammographic modalities such as digital breast tomosynthesis and contrast-enhanced mammography. In these contexts, the integration of deep learning, radiomics, and radiogenomics has shown promise in enhancing diagnostic accuracy and risk stratification [28,40,45].

From an ethical and regulatory standpoint, Rentiya et al. [29] and Hussain et al. [38] emphasize the need for continued attention to validation processes, privacy concerns, bias mitigation, and governance frameworks to ensure safe and responsible AI deployment in clinical practice.

### 4.2. Advances in Breast Cancer Research and Mammographic Technologies: The Role of AI

The field of breast cancer research has experienced significant expansion in recent years, driven by rising incidence rates globally and a growing emphasis on early detection, personalized therapies, and improved patient outcomes. Market analyses indicate that the global breast cancer therapeutics sector was valued at approximately USD 29.85 billion in 2023 and is projected to reach around USD 60.78 billion by 2033, growing at a compound annual growth rate (CAGR) of 7.37% from 2024 to 2033 [49].

Concurrently, advancements in mammographic technologies, particularly digital breast tomosynthesis (DBT), are reshaping breast cancer diagnostics. DBT offers improved cancer detection rates and reduced false positives compared to traditional 2D mammography, leading to increased adoption globally. The global mammography market, encompassing imaging hardware, AI-driven software, and related diagnostic services, was valued at over USD 2.93 billion in 2023 and is expected to expand at a CAGR of approximately 8.68% through 2031 [50,51].

Moreover, the integration of artificial intelligence (AI) tools into mammography is accelerating, supporting enhanced image interpretation, risk assessment, and workflow efficiency in clinical settings. The global AI in medical imaging market was valued at USD 1.29 billion in 2023 and is projected to grow by 22.4% to reach USD 1.65 billion in 2024 [52]. Specifically, AI in the mammography sector is forecasted to increase from USD 509.58 million in 2024 to USD 844.52 million by 2034, growing at a CAGR of 3.3% from 2025 to 2034 [53]. This rapid growth is driven by ongoing improvements in deep learning algorithms, expanding annotated imaging datasets, and greater regulatory approvals for AI-assisted diagnostic tools.

These interrelated sectors—breast cancer therapeutics, mammography technologies, and AI integration—are poised for continued expansion as research advances, regulatory approvals progress, and clinical adoption widens. Together, they highlight a rapidly evolving ecosystem focused on improving early detection, diagnostic accuracy, workflow optimization, and ultimately, patient survival and quality of life [49,50,51,52,53].

### 4.3. Towards Routine Integration: Coordinated Efforts for AI in Mammography

Recent advancements in mammographic technologies combined with the integration of artificial intelligence (AI) are revolutionizing breast cancer diagnostics. However, despite promising developments, several critical challenges must be overcome before AI can be reliably and routinely implemented in clinical practice. Among these challenges, ensuring the quality, representativeness, and diversity of imaging datasets is fundamental to avoid biases and to guarantee algorithmic robustness across different populations and imaging conditions.

Furthermore, AI systems must offer explainability to clinicians to build trust and facilitate transparent diagnostic decisions. Prospective validation in real-world clinical environments remains essential to confirm the clinical utility and safety of these tools. Regulatory frameworks governing AI applications in healthcare are still in a state of flux, requiring clearer guidance to streamline approval and adoption processes while safeguarding patient safety.

Beyond these technical and regulatory concerns, additional factors critically influence the integration of AI in mammography. The impact of AI on radiologist training and continuing education must be carefully considered, as reliance on automated systems could affect the development and maintenance of human diagnostic skills. Moreover, medico-legal responsibilities surrounding AI-assisted diagnoses are complex and need clear definitions to allocate liability appropriately.

Finally, the patient–clinician relationship may evolve with AI involvement. It is imperative to understand how AI influences communication, patient trust, and shared decision-making, ensuring that technology complements rather than compromises the human aspects of care.

Addressing these multifaceted challenges through interdisciplinary research and policy development is vital to harness AI’s full potential in breast cancer screening and diagnosis, ensuring equitable, safe, and effective care.

In the United States, the FDA has released a Draft Guidance on Artificial Intelligence-Enabled Device Software Functions, proposing a Total Product Lifecycle (TPLC) regulatory approach which is directly applicable to imaging-based AI systems, including those used in mammography [54]—aspects crucial to ensure the fairness and robustness of AI tools across diverse populations, addressing concerns about dataset bias and algorithmic transparency. Furthermore, in collaboration with Health Canada and the UK’s MHRA, the FDA introduced the concept of Predetermined Change Control Plans, designed to manage real-world updates to approved AI tools, including those used in breast imaging workflows [55]—highlighting the need for continuous oversight and adaptive regulation in a rapidly evolving AI landscape, which also raises questions about ongoing medico-legal responsibilities. Professional societies such as the American College of Radiology (ACR) have emphasized that AI in mammography should serve as a decision-support tool—augmenting rather than replacing radiologists—particularly in the role of a second reader [56]—reinforcing the importance of maintaining human oversight and considering the impact of AI on radiologist training and clinical workflows.

In the European Union, the Artificial Intelligence Act—effective from August 2024—classifies AI used in mammography as a high-risk medical application. This mandates strict safeguards, including transparent data handling, human oversight, and ongoing post-deployment monitoring [57,58]—measures that directly address ethical standards and patient safety, while also underscoring challenges in integrating AI without disrupting patient–clinician relationships. Supporting these policies, the European Society of Breast Imaging (EUSOBI) has published guidance specific to the safe and effective adoption of AI in breast imaging. Their initiatives—such as the 2025 symposium “Mammography & Beyond: Special Focus on AI”— represent guidance in evolution and reiterreiterate the importance of integrating AI within evidence-based mammography practices [57]—promoting multidisciplinary collaboration to balance technological innovation with clinical realities.

Across North America and Europe, radiology organizations including the ACR, the Canadian Association of Radiologists (CAR), and the EUSOBI are setting standards for AI implementation in mammography. Their guidelines focus on ensuring image quality, enhancing interpretive accuracy, and improving workflow efficiency. Collaborative projects, such as the ACR’s partnership with Densitas, are already contributing to AI-driven quality assurance protocols in mammographic positioning and acquisition [59]—demonstrating practical steps to improve diagnostic performance, yet also requiring consideration of how such tools affect professional roles and responsibilities.

Importantly, the sustained transition from experimental AI tools to routine clinical mammography requires harmonized multistakeholder efforts. Regulatory agencies must maintain flexible yet rigorous approval pathways; scientific societies should generate shared, evidence-based protocols for AI in breast imaging; and large-scale infrastructures such as the UK NHS AI Lab and the European Health Data Space must continue to invest in diverse, multicenter validation initiatives. Notable efforts include the NHS’s prospective evaluation of AI tools in large-scale mammography programs, as well as pilot studies at Imperial College London and Leeds Teaching Hospitals, which test AI integration in symptomatic and screening settings [60]—highlighting the importance of prospective clinical validation and large-scale data to support safe and effective AI adoption.

In parallel, it is essential to address less frequently discussed but equally critical challenges such as the impact of AI on radiologist training pathways, the evolving medico-legal responsibilities surrounding AI-assisted diagnoses, and the shifting dynamics in patient–clinician communication and trust.

Altogether, these coordinated developments reflect a growing consensus: fully realizing the clinical promise of AI-enhanced mammography depends on aligned standards, regulatory maturity, and scientifically guided implementation—ensuring that technological progress ultimately translates into better early detection, diagnosis, and outcomes for patients.

### 4.4. From Promise to Practice: Advancing AI in Mammography Through Trials, Innovation, and Translation

In light of emerging recommendations from systematic reviews—which represent the foundational evidence base of this narrative review—and recent clinical guidelines, it is strategically essential to broaden the scope of analysis by integrating cutting-edge research outputs, especially those arising from prospective clinical trials, randomized controlled trials (RCTs), and the latest studies disseminated through IEEE and ACM conferences and preprint repositories.

The studies included in this analysis were rigorously selected following the search strategy and keyword criteria outlined in Section 2. This approach ensured the inclusion of the most relevant and recent evidence contributing novel insights to the field. Specifically, the selection encompassed systematic reviews and meta-analyses that synthesize current knowledge, alongside prospective clinical trials and RCTs that provide high-level evidence on the clinical efficacy and safety of AI applications in mammography. Additionally, technical papers from IEEE and ACM venues were incorporated to capture recent algorithmic innovations, system design, and implementation challenges that are often not yet reflected in the clinical literature.

This comprehensive integration is crucial for several reasons. First, systematic reviews and clinical guidelines primarily synthesize existing evidence to offer a consolidated understanding of the current state of knowledge, but they often lag behind the most recent technological and methodological innovations due to the inherent delays in publication and guideline updates. Incorporating fresh empirical data from high-quality clinical trials—particularly RCTs—provides robust, prospective, and contextually relevant evidence that can confirm, nuance, or challenge previous conclusions derived from retrospective or more controlled study designs.

Second, studies published in technical forums such as the IEEE and ACM often present novel algorithmic developments, optimization techniques, and real-world implementation insights that are not yet fully captured in traditional systematic reviews. These contributions provide valuable perspectives on practical challenges, including model generalizability across heterogeneous populations and imaging settings, workflow integration, explainability, and human–AI interaction—elements that are critical for successful clinical adoption but frequently under-reported in guideline documents.

Third, preprint platforms accelerate the dissemination of pioneering research, introducing novel approaches and hypotheses that push the boundaries of AI in mammography. Although these studies require cautious interpretation due to the absence of formal peer review, their early availability promotes timely scientific dialog and can inspire rapid advancements and collaborative validation efforts.

By synergistically combining evidence from systematic reviews, guidelines, prospective trials, technical conference papers, and preprints, this narrative discussion gains a multidimensional perspective. Such a comprehensive approach not only strengthens the robustness of the conclusions but also aligns with the complex, interdisciplinary nature of AI integration in breast cancer screening. It underscores the importance of addressing technical performance alongside clinical, organizational, ethical, and regulatory dimensions, ultimately facilitating the translation of AI innovations into safe, effective, and equitable diagnostic tools.

*Bridging Evidence and Practice: What Clinical Trials Reveal About AI in Mammography*

While recent guidelines and systematic reviews have thoroughly outlined the technical, ethical, and regulatory barriers to clinical implementation of AI in mammography, prospective clinical trials and RCTs add essential layers of evidence that either reinforce or nuance these concerns [61,62,63,64,65,66,67,68,69,70,71,72,73]. First, real-world trials offer a unique opportunity to evaluate AI systems on heterogeneous populations, under non-curated conditions, and with clinical endpoints that go beyond model accuracy. Unlike retrospective studies, which often rely on idealized datasets, these trials can expose the fragility of models when applied across different imaging devices, clinical workflows, and patient demographics.

The MASAI trial [61,69], one of the most robust RCTs to date, exemplifies this transition by demonstrating non-inferiority of AI-supported screening compared to standard double reading. Importantly, the AI-assisted arm maintained sensitivity while significantly reducing reader workload, suggesting a promising avenue to alleviate radiologist shortages without compromising diagnostic accuracy. This type of prospective, population-based evaluation is a crucial milestone toward clinical translation, particularly in settings where human resource constraints threaten the scalability of breast screening programs.

Notably, some RCTs confirm the problem of generalizability, revealing performance drops when AI is tested outside its original training domain—thereby substantiating concerns raised in systematic reviews about dataset representativeness and algorithmic bias [24,25]. For example, while studies like Dembrower et al. [68] and Salim et al. [65] confirmed the high concordance of AI predictions with radiologist assessments in Swedish and Nordic cohorts, similar results are not guaranteed in more diverse or resource-constrained settings. Here, the role of trials is not merely to reproduce findings but to stress-test them under real-world variability.

Moreover, hybrid AI–radiologist workflows, although sometimes associated with improved accuracy, have been found to increase recall rates and workload, potentially leading to overdiagnosis—subtleties that are often overlooked in traditional benchmarking studies. This highlights the importance of outcome measures that extend beyond sensitivity and specificity, incorporating downstream clinical and organizational impacts.

Clinical trials also bring to light pragmatic insights on explainability and usability. While many systematic reviews emphasize the “black box” nature of deep learning, empirical data reveal that explainability tools (e.g., heatmaps or saliency maps) are frequently underutilized or misunderstood by clinicians, limiting their real-world effectiveness. Similarly, integrating AI into existing workflows entails logistical and cognitive burdens not always captured by technical assessments. The multireader study by Rangarajan et al. [64] underscores this by evaluating how AI tools function in educational contexts, offering insights into human–machine interaction beyond diagnostic metrics.

From a regulatory standpoint, trials help inform nuanced risk–benefit analyses and reveal the lack of context-specific evaluation frameworks. Even high-performing AI systems may falter in adoption due to documentation gaps, misalignment with health IT infrastructures, or lack of interoperability standards. In this sense, trials can act as a bridge between laboratory performance and regulatory readiness, identifying implementation bottlenecks early.

Emerging directions such as radiomics-based personalization [67,70] and AI-driven risk stratification [65] show promise in tailoring screening protocols. Yet, these approaches remain at the feasibility stage, and longitudinal outcome data are still scarce. Moreover, the methodological heterogeneity across trials—ranging from prospective paired-reader designs to retrospective validations—makes it difficult to synthesize findings in a standardized way. This calls for harmonized reporting standards and multicenter collaborations to enable meaningful meta-analyses.

Overall, prospective trials and RCTs not only validate or contest prior findings from systematic reviews and guidelines but also shift the conversation toward operational, behavioral, and implementation-related challenges. Bridging the gap between AI potential and clinical trustworthiness requires interdisciplinary collaboration, robust prospective evidence, and attention to the social and organizational dimensions of care.

Based on the current evidence, we recommend several key priorities to accelerate the safe and equitable integration of AI into mammography screening. First, expanding prospective multicenter trials across diverse healthcare systems and patient populations is essential to improve the generalizability of AI models and reduce potential biases. It is also important to incorporate outcome measures that go beyond simple accuracy metrics, including recall rates, workflow efficiency, clinician trust, and patient experience, to fully capture AI’s impact in clinical practice.

The standardization of explainability tools and comprehensive clinician training must be prioritized to ensure that healthcare professionals can reliably interpret and trust AI outputs during diagnostic workflows. Furthermore, regulatory alignment should be strengthened by designing clinical trials that proactively address documentation requirements and interoperability standards, facilitating smoother adoption and oversight. Finally, fostering interdisciplinary partnerships among AI developers, clinicians, patients, and policy-makers is critical to ensure that technological innovations are closely aligned with real-world clinical needs and ethical considerations.

Ultimately, clinical trials must evolve from being post hoc validations to becoming proactive instruments of AI co-design, contextualization, and continuous learning in mammographic care.

*Next-Gen AI for Mammography: Multimodal Learning and Optimization Strategies*

To complement existing systematic reviews and clinical guidelines, recent articles from the ACM Digital Library provide cutting-edge algorithmic innovations and optimization strategies that address practical challenges in mammography AI, offering fresh empirical evidence and highlighting diverse methodological approaches.

Recent advancements in AI-driven mammography diagnosis demonstrate significant progress in addressing the complex challenges of early breast cancer detection, with recent studies exploring multi-granularity, optimization-based, and YOLO-based approaches [74,75,76], which exemplify different strategies for improving AI-based mammography diagnosis.

Wei and Fan [74] propose a multi-granularity, knowledge-guided multimodal pre-training approach (MM-CLIP) specifically tailored to breast cancer diagnosis. Their method stands out by effectively focusing on the small, complex lesion areas that distinguish mammogram analysis from conventional natural image recognition. By capturing diverse lesion morphologies and accounting for anatomical variability, this approach enhances diagnostic accuracy beyond prior CLIP-based models, marking a promising direction for multimodal AI integration in clinical mammography.

Complementing this, Doma et al. [75] leverage Weighted Particle Swarm Optimization (WPSO) to optimize the extraction of texture features from segmented mammogram images. This approach specifically targets microcalcifications—a critical early sign of breast cancer—enabling more precise classification into normal, benign, or malignant categories. Their AI framework reduces manual diagnostic overhead and supports the rapid detection of cancerous regions in hospital-derived mammogram datasets, highlighting the value of evolutionary optimization techniques in improving diagnostic efficiency and accuracy.

Further advancing the field, using a YOLOv5-based CAD framework, breast masses were detected and classified across multiple datasets, including CBIS-DDSM, VinDr-Mammo, and INBreast, as shown by Aydemir et al. [76]. The approach employed transfer learning and data augmentation to enhance accuracy and generalizability. Fivefold cross-validation yielded mAP around 0.84, with precision up to 0.855 and recall up to 0.787. Testing on MIAS and private clinical datasets confirmed the model’s potential to support radiologists in reliable and efficient breast cancer diagnosis [76]. Overall, these studies highlight several critical themes for the application of artificial intelligence in mammography. First, there is a clear need to focus on fine-grained, lesion-level information through multi-modal and multi-granularity models to capture the complexity inherent in mammographic images. Additionally, optimization algorithms such as WPSO and Coati play a crucial role in enhancing feature extraction and selection, which directly impacts diagnostic accuracy. Another key advantage comes from hybrid architectures that combine localization, feature extraction, and robust classification to effectively manage the variability and complexity of mammographic data.

Looking ahead, future research and clinical deployment should prioritize further multimodal integration, combining imaging data with clinical and genetic information to develop more contextualized and precise diagnostic models. The prospective validation of these optimized AI models in diverse, real-world clinical settings is essential to ensure their generalizability and operational feasibility. Emphasis must also be placed on interpretability and clinician usability, making sure that AI outputs are actionable and trusted within clinical workflows. Lastly, the development of standardized benchmarking frameworks that incorporate lesion complexity and heterogeneity will be necessary to rigorously compare emerging AI methods and foster progress in the field.

By addressing these challenges, the field can accelerate the transition from promising algorithms to clinically reliable tools that improve breast cancer diagnosis and patient care.

*From Lab to Clinic: Usability and Integration Lessons from IEEE Research*

A glance at IEEE conference papers offers valuable additions to the knowledge base established by systematic reviews and clinical guidelines by emphasizing technological innovation and practical AI applications in mammography. While systematic reviews and guidelines primarily synthesize and standardize existing evidence to guide clinical practice, IEEE research frequently explores novel methodological approaches and addresses challenges in real-world deployment.

For instance, Islam et al. [77] demonstrate a significant advancement in breast cancer detection accuracy by applying a deep learning approach to the recently released RSNA Screening Mammography dataset. Their study goes beyond prior work by focusing in detail on image preprocessing techniques and the critical factor of breast density, which is often overlooked. By investigating these aspects, they reveal practical ways to enhance AI model performance, achieving a high classification accuracy of 97.6% using a tailored 15-layer CNN. This granular attention to imaging nuances offers a valuable complement to the broader recommendations found in guidelines.

Yang et al. [78] contribute by developing MABEL, an AI-powered mammographic lesion diagnostic system that is specifically designed to integrate seamlessly into clinical workflows. Their system not only reduces radiologists’ workload but also accelerates diagnosis through an intuitive doctor-oriented annotation tool and web interface. Furthermore, MABEL’s compatibility with hospital Picture Archiving and Communication Systems (PACSs) reflects a mature stage of translational research, emphasizing the importance of usability, scalability, and real-world application—factors that systematic reviews and clinical guidelines typically do not address in depth.

Similarly, Rath et al. [79] provide a comprehensive empirical comparison of multiple AI algorithms and preprocessing strategies for mammogram analysis. By assessing various classification models including SVMs, KNNs, Random Forest, Decision Trees, CNNs, and RCNs, they identify the strengths and limitations of each approach in practical scenarios. Their exploration of image segmentation and feature extraction methods further enriches the understanding of how preprocessing influences diagnostic accuracy. This type of comparative research informs clinicians and developers about optimal AI solutions for implementation, supplementing the evidence summaries provided by systematic reviews.

Together, these IEEE studies exemplify how conference research can push the field forward by introducing innovative methodologies, addressing practical clinical integration challenges, and providing empirical data to guide AI deployment decisions. This approach offers a dynamic perspective on advancing AI-assisted breast cancer diagnosis beyond the scope of traditional evidence synthesis.

*The Frontier of Innovation: Emerging Trends in Preprints on AI for Mammography*

While peer-reviewed publications remain the cornerstone for establishing reliable evidence and clinical guidelines, recent preprints available on open repositories like arXiv are increasingly shaping the forefront of AI research in mammography. These preprints serve as a valuable window into the very latest innovations and exploratory methodologies, often addressing topics and challenges not yet fully covered in the formal literature. However, it is crucial to interpret their findings with appropriate caution since these studies have not yet undergone thorough peer review or clinical validation, processes essential for confirming reproducibility, robustness, and safety in real-world applications.

The preprints highlighted here reveal several critical and very hot themes currently driving the field forward. For instance, the study by [80] introduces advanced deep learning architectures that aim to significantly improve the precision of lesion detection and characterization. These models incorporate novel feature extraction layers designed to better capture subtle imaging patterns indicative of malignancy. In parallel, ref. [81] explores the integration of clinical metadata—such as patient history and genetic markers—with imaging data, aiming to build more contextualized and personalized diagnostic AI models. This multimodal approach acknowledges the complexity of breast cancer diagnosis beyond image pixels alone.

Additionally, ref. [82] focuses on enhancing the interpretability of AI decisions through innovative attention mechanisms and explainability tools. Given that clinical adoption strongly depends on trust and transparency, such advances are critical to bridging the gap between black-box AI systems and radiologists’ workflow. Finally, ref. [83] addresses the challenge of scalability by proposing efficient training strategies that allow AI models to be trained on large, heterogeneous mammography datasets, ensuring broader applicability across diverse populations and imaging devices.

Together, these preprints highlight exciting, forward-looking directions for AI in mammography, ranging from methodological innovation to practical deployment considerations. Nonetheless, to translate these promising research advances into safe and effective clinical tools, it is essential that future work rigorously validate these models through peer review, prospective clinical trials, and real-world implementation studies.

Therefore, we recommend that the AI research community maintain a balanced approach: continue to foster innovation by sharing and discussing preprint findings openly while prioritizing robust validation and interdisciplinary collaboration with clinicians. This pathway will help ensure that novel AI technologies are not only cutting-edge but also trustworthy, equitable, and ultimately beneficial for patient care.

### 4.5. Limitations

This narrative review synthesizes peer-reviewed systematic reviews and meta-analyses on artificial intelligence (AI) applications in mammography using a structured yet flexible and transparent methodology. Rather than adhering strictly to the format of a fully systematic review, we adopted a narrative review approach to better accommodate the rapidly evolving and highly diverse nature of this field, where AI techniques, clinical applications, and study designs vary widely.

This narrative approach enabled us to capture emerging trends and novel applications of AI that might be overlooked by more rigid frameworks, providing a timely and interpretative synthesis of the current state of the art. While this method does not follow exhaustive search protocols or formal risk-of-bias assessments typical of systematic reviews, we ensured methodological rigor by applying a standardized quality checklist to guide study selection and appraisal, maintaining internal consistency and reliability in the included evidence.

Our literature search focused on major biomedical databases including PubMed, Scopus, and the Web of Science, providing comprehensive coverage of systematic reviews and meta-analyses. To complement this core evidence base, we also incorporated findings and recommendations from clinical practice guidelines, randomized controlled trials (RCTs), and other prospective clinical studies. Additionally, databases such as the IEEE Xplore and ACM Digital Library, as well as preprint servers, were consulted to include the most recent and technologically oriented research outputs. This complementary approach enriched our synthesis by integrating high-level evidence and expert consensus alongside emerging clinical data.

Only English-language publications were included to maintain feasibility, which may have excluded some valuable regional or non-English research. Although access to scientific outputs from radiological and oncological societies in original languages would add further depth, the challenges related to comprehensive retrieval and translation exceed the scope of this broad narrative review and suggest an avenue for future focused studies.

In summary, this review provides a comprehensive and balanced overview of AI’s role in mammography screening, highlighting its promising potential supported by a growing and varied evidence base. The integration of systematic reviews, clinical guidelines, RCTs, and other clinical studies forms a robust foundation to inform future research directions, clinical implementation strategies, and policy-making. This collective evidence supports cautious optimism toward AI adoption while emphasizing the need for further high-quality, prospective, multicenter trials to confirm effectiveness and safety in diverse clinical settings.

## 5. A Final Thought: Beyond the Hype—Unlocking the True Clinical Potential of AI in Mammography Through Multidimensional Evidence

Based on the comprehensive findings of the systematic review and complemented by clinical guidelines, randomized controlled trials (RCTs), prospective studies, emerging methodological innovations, and real-world implementation research, the future of artificial intelligence applied to mammography requires a multidimensional approach that goes well beyond mere technical validation, encompassing clinical, organizational, ethical, and regulatory aspects. The following strategic priorities can be outlined.

Expansion of multicenter prospective clinical trials

To improve the generalizability and robustness of AI models, it is essential to conduct studies on heterogeneous populations in real-world conditions that reflect the diversity of devices, clinical environments, and demographic groups. Only large-scale randomized controlled trials (RCTs) can confirm AI’s efficacy and safety compared to established practices, reducing sampling bias and overcoming the limitations of retrospective analyses. *These trials should also address medico-legal responsibilities and accountability in diagnostic decision-making, ensuring clarity on the role of radiologists and AI tools within clinical workflows.*

Adoption of broader clinical and organizational metrics

Traditional diagnostic accuracy measures (sensitivity and specificity) should be integrated with indicators of clinical and organizational impact, such as recall rates, overdiagnosis, radiologist workload, clinician confidence, and patient experience. These endpoints allow the evaluation of AI’s real effects on care pathways, avoiding unwanted consequences such as overload or increased false positives. *Importantly, patient-centered outcomes and changes in the patient–clinician relationship must be assessed to preserve trust and shared decision-making.*

Standardization and training on explainability

Explainability tools (e.g., heatmaps) must be standardized and incorporated into dedicated clinical training programs so healthcare professionals can correctly interpret and effectively use them in decision-making, reducing the perception of a “black box” and improving trust in AI. *Enhancing interpretability directly supports radiologist training and mitigates concerns over reliance on opaque algorithms.*

Integration with healthcare systems and interoperability

Clinical trials should include assessments of integration requirements with existing IT infrastructures such as PACSs and electronic health records, as well as adherence to regulatory standards. The lack of interoperability and complete documentation remains a crucial barrier to large-scale clinical adoption. *Moreover, potential disruptions to workflow, increased cognitive load, and risks of clinician burnout must be carefully managed.*

Development and validation of multimodal and personalized models

The future of AI in mammography will move from image-only models to multimodal systems that integrate clinical, genetic, and radiomic data for more precise and personalized diagnosis and risk stratification. These models require rigorous prospective validation and large longitudinal studies to confirm effectiveness and sustainability. *Ethical considerations, including data privacy, informed consent, and equitable access, become increasingly critical as these models incorporate sensitive patient information.*

Algorithmic optimization with evolutionary and hybrid methods

The adoption of advanced optimization techniques (e.g., Particle Swarm Optimization, Coati Optimization) and hybrid architectures (e.g., YOLOv7 + UNet) could represent an important direction to improve feature extraction, lesion localization, and classification, increasing accuracy and clinical reliability. *However, greater algorithmic complexity heightens the need for explainability and regulatory scrutiny.*

*Research on hybrid workflows and human–machine interaction*

New studies should explore how workflows integrating AI and radiologists affect not only diagnostic outcomes but also clinical organization and recall decisions. Under-standing cognitive and organizational dynamics is essential to avoid overload and ensure effective and sustainable AI use. *This includes clarifying roles and responsibilities and understanding impacts on patient communication and shared decision-making.*

Interdisciplinary collaboration and stakeholder engagement

The development, validation, and implementation of AI in mammography require close collaboration among developers, clinicians, patients, regulators, and policymakers. This co-design approach fosters technological solutions that address real clinical needs, are ethically sustainable, and facilitate adoption in diverse healthcare settings. *The early engagement of patients and clinicians helps build trust and align expectations.*

Adoption of standardized reporting and benchmarking frameworks

To enable objective comparisons and shared progress, common standards are needed for reporting clinical trial results and performance studies, accounting for lesion complexity, data heterogeneity, and population variability. *Transparent reporting supports regulatory oversight and addresses reproducibility concerns.*

From post hoc validations to trials as tools for co-design and continuous learning

Clinical trials must evolve from simple validation tools to dynamic platforms for the co-design, adaptation, and continuous monitoring of AI performance, promoting constant model updates based on real-world data and clinical feedback. *This dynamic approach is crucial for managing algorithm updates and maintaining compliance with evolving ethical and legal frameworks.*

Overall, the transition from the “promised AI” to “clinical practice” in mammography requires a holistic and integrated approach that combines robust prospective evidence from systematic reviews, clinical guidelines, RCTs, and real-world studies with algorithmic innovation, usability focus, and strong interdisciplinary collaboration. *Addressing training needs, medico-legal challenges, organizational changes, and patient–clinician dynamics is essential to develop AI diagnostic tools that are reliable, safe, sustainable, and truly beneficial for patients.*

## 6. Conclusions

This narrative review is grounded in a comprehensive analysis of 28 systematic reviews and meta-analyses that evaluate the role of artificial intelligence (AI) in mammography. These high-level evidence syntheses collectively highlight a promising potential for AI technologies to improve diagnostic accuracy, sensitivity, and specificity in breast cancer screening compared to conventional methods. The integration of AI-assisted tools demonstrates encouraging advancements in lesion detection, reductions in false positives, and improved workflow efficiency. Complementing these syntheses, this review also examined available randomized controlled trials (RCTs) and other clinical studies investigating AI applications in mammographic screening. Although these prospective studies provide initial empirical support for AI’s clinical utility, they remain limited in number, sample size, and methodological rigor. Many clinical investigations suffer from short follow-up durations, inconsistent outcome measures, and varying definitions of performance metrics, which restrict the generalizability of their findings. Moreover, a significant challenge identified across the evidence base is the heterogeneity of study designs, AI algorithms, datasets, and population characteristics. Retrospective data predominates the field, often lacking standardized protocols and blinded assessments, which can introduce bias and limit reproducibility. The variability in AI model training and validation strategies further complicates direct comparisons and the aggregation of results. Given these limitations, the current body of evidence, while promising, is insufficient to support broad implementation of AI systems in routine mammographic screening. There is a pressing need for rigorously designed, large-scale, multicenter prospective trials that adopt harmonized methodologies and standardized outcome reporting. Such studies are essential to accurately assess AI’s clinical impact, safety, and cost-effectiveness across diverse healthcare settings. In addition to clinical validation, future research should also address ethical considerations, data privacy, and the practical challenges of integrating AI tools within existing diagnostic workflows. Transparency in AI decision-making processes and user training will be critical to foster clinician trust and optimize patient outcomes. In summary, this narrative review underscores AI’s potential to transform breast cancer screening but advocates for a cautious and evidence-driven approach. Prioritizing high-quality prospective research and comprehensive evaluation frameworks will enable the responsible adoption of AI technologies, ensuring they deliver genuine clinical benefit while minimizing risks and disparities.

## Figures and Tables

**Table 1:** Common themes identified across included systematic reviews.

Common Theme	Summary	Key References
Reader cancer detection comparison	Most systematic reviews explore AI systems that show comparable sensitivity and specificity to radiologists in mammography and suggest they are an advanced technique for cancer detection.	[21,22,24,29,31,32,34,35,36,40,41,42,43,44,45,46,48]
Radiologist workload reduction	AI can triage low-risk mammograms, thereby reducing the number of images requiring human interpretation without compromising sensitivity.	[21,30,31]
Error types	AI errors are typically quantified in terms of false positives and negatives, which vary based on positivity thresholds, algorithm version, and study quality.	[23]
Ethical implication	Many studies explore the ethical implication, challenges and frameworks on the use of AI software in clinical practice.	[29,36,37]
Methodological limitations	Many studies are retrospective, with concerns about bias, limited generalizability, and the inconsistent reporting of accuracy metrics, highlighting the necessity of performing more rigorous prospective studies.	[24,29,32,33,34,45,47]
Combined AI and radiologist performance	The integration of AI with radiologist interpretation frequently results in improved diagnostic accuracy compared to using radiologists alone.	[26,34,35]
Prospective and future applications	AI technology shows promise for future risk-based screening, early detection, and personalized screening strategies based on imaging features.	[27,29,37,38,44]
Performance on advanced imaging technique	AI has been applied to modalities such as DBT and CEM, showing promising yet preliminary results.	[22,38,40,45]

AI: artificial intelligence; CEM: contrast-enhanced mammography; DBT: digital breast tomosynthesis.

**Table 2:** Insights on the use of AI and on technological integration.

Ref.	Brief Description	AI Applications and Technical Details
[21]	Systematic review on AI in screening mammography focusing on diagnostic accuracy, reductions in false positives, and radiologist support. Analyzes 13 studies on AI performance in breast cancer detection.	AI algorithms assist in mammography readings, sometimes outperforming double radiologist readings. Techniques improve specificity, reduce unnecessary recalls, and lower workload. Challenges include false positives and variation in performance across demographics.
[22]	Meta-analysis assessing standalone AI performance in digital mammography and digital breast tomosynthesis (DBT) screening. Includes 16 studies with over 1 million exams.	Standalone AI evaluated via reader and cohort studies. AI shows higher AUC than radiologists in digital mammography and DBT. AI demonstrates higher sensitivity but lower specificity compared to radiologists. Uses QUADAS-2 for study quality assessment.
[23]	Systematic review focusing on error types and frequencies in AI mammography readings, analyzing false-positive and false-negative rates across 7 retrospective studies.	Errors analyzed include false positives and false negatives varying by AI threshold and algorithm version. Meta-analysis of pooled error rates conducted. Sparse reporting of other AI error types such as location or technical errors.
[24]	Systematic review of AI test accuracy in breast cancer screening programs including 12 studies (131,822 women). Examines AI alone or combined with radiologists.	Most AI systems less accurate than single radiologists and far less than double readings. Some small studies show promise in lab settings. AI used for triage screens out low-risk women but can miss some cancers. No prospective clinical studies found. QUADAS-2 tool used for quality assessment.
[25]	Review of mammography datasets used in AI development, focusing on dataset diversity, inclusivity, traceability, and accessibility.	Identifies 254 datasets with most privately held; poor demographic reporting (race/ethnicity, sex/gender). Highlights global representation gaps and documentation transparency issues, impacting AI model generalizability and fairness.
[26]	Systematic review of external validation studies for AI algorithms in mammography breast cancer screening (30 studies, 2014–2024).	AI algorithms demonstrate comparable AUC and sensitivity to radiologists alone. Combining AI with radiologists improves accuracy statistically. Studies include diverse populations and validation datasets. Emphasizes need for external validation.
[27]	Systematic review on integration of AI in breast cancer screening workflows, focusing on clinical implementation challenges and real-world performance.	Discusses AI deployment in clinical settings, highlighting variability in algorithm performance, integration with radiologists, regulatory considerations, and workflow optimization. Emphasizes importance of prospective studies and continuous monitoring of AI tools.
[28]	Systematic review of AI applied to medical imaging research in Italy (2015–2020). Main imaging modalities: MRI (44%), CT (12%), radiography/mammography (11%). Focuses on neurological diseases (29%) and cancer diagnosis (25%). AI tasks: classification (57%), segmentation (16%). Methods: 65% machine learning, 35% deep learning. Rapid growth in AI research observed.	AI used primarily for image classification and segmentation across multiple imaging modalities. Machine learning and deep learning models analyzed. Emphasis on building common frameworks, data sharing, and collaborations.
[29]	Review of AI advancements in breast cancer detection and treatment, highlighting shift from CAD to AI-based methods. Discusses challenges like data quality, regulation, ethics, and validation. Promising potential to improve early diagnosis and patient outcomes.	AI applications in automated interpretation of mammograms and tomosynthesis. Deep learning methods improve accuracy and efficiency in detection. Challenges include algorithm validation and clinical integration.
[30]	Systematic review of AI in mammography focusing on diagnosis and prediction of breast malignancies. Reports reduction in false positives (up to 69%) and increased sensitivity (84–91%). AI models can independently classify suspicious findings comparable to radiologists.	Machine learning models applied to reduce radiologist workload, improve diagnosis accuracy, and predict breast cancer risk. Calls for larger prospective studies to confirm clinical utility.
[31]	Meta-analysis on deep learning (DL) software for triaging breast cancer screening mammograms. Shows 68.3% reduction in radiologist workload with 93.1% sensitivity. Highlights complexity of AI implementation but promising healthcare optimization.	DL algorithms used for triage of mammograms to exclude low-risk cases, maintaining high sensitivity. Meta-analysis of commercially available DL software with performance metrics reported.
[32]	Systematic review and meta-analysis of standalone machine learning (ML) algorithms for screening mammography workflow. ML algorithms achieve or exceed human reader performance with pooled sensitivity of 75.4%, specificity of 90.6%, AUC of 0.89.	Standalone ML models for mammogram detection and triage, evaluated independently from human readers. Evidence based on retrospective studies; external prospective validation needed.
[33]	Meta-analysis of ML methods (CNN, ANN, SVM) in mammography diagnosis for breast cancer screening. CNN showed highest sensitivity (96.1%), specificity (95.0%) and AUC (0.974). Emphasizes need for prospective studies.	Evaluation of different ML methods for breast cancer diagnosis on mammography images. CNNs demonstrate superior performance compared to ANN and SVM.
[34]	Systematic review of independent external validation studies of AI algorithms for screening mammography. Some AI algorithms improve accuracy over radiologists alone; combined AI and radiologist interpretation shows further improvement.	External validation of commercial AI tools for mammography cancer detection. Studies include retrospective reader and simulation designs. AI improves diagnostic accuracy, especially when combined with radiologists.
[35]	Systematic review evaluating reproducibility and explainability of deep learning in mammography for breast cancer detection. Found high risk of bias in most studies; only few of adequate quality. Common architectures include ResNet and RetinaNet. Highest AUC reported was ~0.945. Combined AI and radiologist readings improve performance. Lack of explainability and real-world interaction studies noted.	Analysis of deep learning models’ reproducibility and clinical validity. Emphasis on need for explainable AI and real-world validation. Ensemble models and patch classifiers common. Highest diagnostic performance when AI assists radiologists.
[36]	Systematic review identifying challenges in AI implementation for breast cancer screening. Key issues: reproducibility, evidentiary standards, tech integration, trust, ethics, legal and societal concerns. Uses CFIR framework to propose governance strategies.	Mainly AI in mammography (19 studies) and ultrasound (1 study). Highlights needs for robust evidence, trust-building, addressing ethical/legal aspects, and structured governance. CFIR helps map challenges to solutions for clinical adoption.
[37]	Review of explainable AI (XAI) methods applied to breast cancer diagnosis via mammography and ultrasound. Investigates evaluation methods, ethical challenges, and trust in XAI systems.	Analyzes 14 studies on XAI for breast cancer imaging. Finds limited evaluation of user trust and confidence. Dataset quality and related issues highlighted as key research gaps. Calls for systematic evaluation of XAI trustworthiness in clinical settings.
[38]	Systematic review on machine learning and deep learning for breast cancer risk prediction using imaging, radiomics, genomics, and clinical data. Discusses current approaches and future potential.	Covers 20 studies using DL models for personalized risk prediction integrating multi-modal data. Explores NLP applications on imaging and non-imaging features to improve clinical decision-making. Provides overview for researchers on AI risk assessment techniques.
[39]	Systematic review and meta-analysis on radiomics to differentiate benign and malignant breast lesions. Assesses diagnostic accuracy across imaging modalities including MRI, mammography, ultrasound, and CT.	Includes 31 studies with 8773 patients. Radiomics showed high sensitivity and specificity across MRI, mammography, and ultrasound, with CT data limited. Supports radiomics as promising adjunct or alternative diagnostic tool, while biopsy remains gold standard.
[40]	Comprehensive review of deep learning, radiomics, and radiogenomics applications in digital breast tomosynthesis (DBT). Focus on DBT’s potential for enhanced early breast cancer detection.	Thirty studies reviewed on DL, radiomics, and radiogenomics applied to DBT, synthetic mammography, and full-field digital mammography. Emphasizes interdisciplinary approaches and new model development for clinical deployment of AI in DBT imaging.
[41]	Systematic review of deep learning in breast cancer detection using multiple imaging modalities. Discusses limitations of current manual analysis and benefits of AI-assisted interpretation.	Reviews latest AI and DL models across mammograms, ultrasound, MRI, and histopathology images. Reports on datasets and algorithm development supporting early detection and improved diagnostic accuracy. Highlights AI’s role in reducing false positives and improving efficiency.
[42]	Systematic review of ML and DL for breast cancer detection using mammographic data. Highlights evolution from traditional ML to deep learning improving CAD systems.	Deep learning methods (CNNs, deep feature learning) enhance diagnostic accuracy in breast cancer CAD; issues include data scarcity and computational cost, mitigated by data augmentation and improved DL architectures.
[43]	Systematic review of machine learning CAD systems for breast cancer using various imaging modalities.	Analysis of ML classifiers and image modalities for breast cancer detection; discussion on improving CAD systems objectivity and efficiency.
[44]	Review on infrared breast thermography for early breast cancer detection, covering image acquisition, segmentation, feature extraction, and classification.	Use of artificial neural networks to improve thermographic image classification; numerical simulation to reduce false positives; ML techniques for real-time applications recommended.
[45]	Systematic review on deep learning applications in contrast-enhanced mammography (CEM).	CNN models dominate; used for lesion classification, detection, segmentation; attention mechanisms improve accuracy; integration with radiomics and radiologist assessments enhances diagnostic performance; mostly retrospective studies, need for prospective validation.
[46]	Meta-analysis of AI-assisted mammography and prognostic role of Systemic Immune–Inflammation Index (SII) in breast cancer.	AI shows high diagnostic accuracy (AUC up to 0.93), reduces radiologist workload and improves detection in dense breasts. SII is prognostic biomarker linked to survival. Challenges include cut-off variability and need for prospective studies.
[47]	Systematic review on imaging modalities for cancer of unknown primary (CUP).	Use of CT, MRI, FDG-PET/CT, emerging whole-body MRI, FAPI-PET/CT, AI and radiomics to improve detection and characterization of primary tumors; evolving imaging tech increases diagnostic precision.
[48]	Systematic review update on breast cancer screening informing Canadian guidelines. Although not focused solely on mammography, this study offers important updated evidence on breast cancer screening, helping to contextualize AI’s role within current screening guidelines.	Machine learning employed to prioritize screening literature; synthesis of RCT and observational data on screening benefits and harms; ML used in research prioritization.

## Data Availability

Not applicable.

## Supplementary Materials

The following supporting information can be downloaded at https://www.mdpi.com/article/10.3390/diagnostics15172197/s1, Table S1. Outcome of the assessment. Box S1. Used search keys in Section 3.1.
