# Supplementary material for: Transforming Breast Imaging: A Narrative Review of Systematic Evidence on Artificial Intelligence in Mammographic Practice
Source: Diagnostics (Basel). 2025 Aug 29;15(17):2197. doi: 10.3390/diagnostics15172197 (PMC12427953; doi:10.3390/diagnostics15172197)
Supplement: Supplementary file 1 [file diagnostics-15-02197-s001.zip › diagnostics-3785212-supplementary.pdf]

Supplementary material of review

# Transforming Breast Imaging: A narrative umbrella review on Artificial Intelligence in Mammographic Practice

**Andrea Lastrucci[1], Nicola Iosca[1], Yannick Wandael[1], Angelo Barra[1], Renzo Ricci[1], Jacopo Nori[2], Nevio Forini[3], Graziano Lepri[4], Daniele Giansanti [5,*]**

1 Department of Allied Health Professions, Azienda Ospedaliero-Universitaria Careggi, 50134 Florence, Italy
2 Department of Radiology, Breast Imaging Unit, Azienda Ospedaliero-Universitaria Careggi, Florence, Italy
3 Dipartimento di Medicina e Chirurgia, Universita' degli Studi di Perugia, Piazzale Settimio Gambuli, 1, 06129 Perugia, Italy
4 Unità Sanitaria Locale Umbria 1, Via Guerriero Guerra 21, 06127 Perugia, Italy
5 Centre TISP, Istituto Superiore di Sanità, 00161 Rome, Italy
* Correspondence: Daniele.giansanti@iss.it

**Table S1.** Outcome of the assessment

| Id | N1 | N2 | N3 | N4 | N5 | N6 |
|---|---|---|---|---|---|---|
| 1 | 5 | 3 | 4 | 3 | 5 | Y |
| 2 | 3 | 4 | 3 | 5 | 4 | Y |
| 3 | 4 | 5 | 5 | 4 | 3 | Y |
| 4 | 3 | 3 | 4 | 5 | 5 | Y |
| 5 | 5 | 4 | 5 | 3 | 4 | Y |
| 6 | 4 | 5 | 3 | 4 | 5 | Y |
| 7 | 3 | 4 | 5 | 5 | 3 | Y |
| 8 | 5 | 5 | 4 | 3 | 4 | Y |
| 9 | 4 | 3 | 3 | 4 | 5 | Y |
| 10 | 3 | 4 | 4 | 5 | 5 | Y |
| 11 | 4 | 5 | 3 | 5 | 3 | Y |
| 12 | 5 | 3 | 4 | 4 | 4 | Y |
| 13 | 3 | 5 | 5 | 3 | 4 | Y |
| 14 | 5 | 4 | 3 | 4 | 5 | Y |
| 15 | 4 | 3 | 5 | 5 | 3 | Y |
| 16 | 3 | 5 | 4 | 3 | 5 | Y |
| 17 | 5 | 4 | 5 | 4 | 4 | Y |
| 18 | 4 | 5 | 3 | 5 | 3 | Y |
| 19 | 3 | 3 | 4 | 4 | 5 | Y |
| 20 | 5 | 4 | 4 | 3 | 4 | Y |
| 21 | 4 | 3 | 5 | 5 | 3 | Y |
| 22 | 3 | 4 | 3 | 4 | 5 | Y |
| 23 | 5 | 5 | 4 | 5 | 4 | Y |
| 24 | 4 | 3 | 5 | 4 | 3 | Y |
| 25 | 3 | 5 | 3 | 3 | 4 | Y |
| 26 | 5 | 4 | 5 | 5 | 5 | Y |
| 27 | 4 | 5 | 4 | 3 | 4 | Y |
| 28 | 3 | 3 | 5 | 4 | 3 | Y |

**Box S1.** Used search keys in section 3.1

| |
|---|
| *(mammography) AND (artificial intelligence)* |
| *(radiology) AND (artificial intelligence)* |
| *(oncology) AND (artificial intelligence)* |
| *(breast cancer) AND (artificial intelligence)* |
